# Superparamagnetic Iron Oxide Nanoparticles and Essential Oils: A New Tool for Biological Applications

**DOI:** 10.3390/ijms21186633

**Published:** 2020-09-10

**Authors:** Maria Graça Miguel, João Paulo Lourenço, Maria Leonor Faleiro

**Affiliations:** 1 Mediterranean Institute for Agriculture, Environment and Development, Faculty of Science and Technology, University of Algarve, Campus de Gambelas, 8005-139 Faro, Portugal; 2 Centro de Química Estrutural, Instituto Superior Técnico, Universidade de Lisboa, Av. Rovisco Pais, 1049-001 Lisboa, Portugal; jlouren@ualg.pt; 3 Centro de Investigação em Química do Algarve (CIQA), Departamento de Química e Farmácia, Faculdade de Ciências e Tecnologia, Universidade do Algarve, Campus de Gambelas, 8005-139 Faro, Portugal; 4 CBMR, Algarve Biomedical Center, Faculdade de Ciências e Tecnologia, Universidade do Algarve, Campus de Gambelas, 8005-139 Faro, Portugal; mfaleiro@ualg.pt

**Keywords:** superparamagnetic iron oxide nanoparticles, synthesis, stabilization, antimicrobial, antibiofilm

## Abstract

Essential oils are complex mixtures of volatile compounds with diverse biological properties. Antimicrobial activity has been attributed to the essential oils as well as their capacity to prevent pathogenic microorganisms from forming biofilms. The search of compounds or methodologies with this capacity is of great importance due to the fact that the adherence of these pathogenic microorganisms to surfaces largely contributes to antibiotic resistance. Superparamagnetic iron oxide nanoparticles have been assayed for diverse biomedical applications due to their biocompatibility and low toxicity. Several methods have been developed in order to obtain functionalized magnetite nanoparticles with adequate size, shape, size distribution, surface, and magnetic properties for medical applications. Essential oils have been evaluated as modifiers of the surface magnetite nanoparticles for improving their stabilization but particularly to prevent the growth of microorganisms. This review aims to provide an overview on the current knowledge about the use of superparamagnetic iron oxide nanoparticles and essential oils on the prevention of microbial adherence and consequent biofilm formation with the goal of being applied on the surface of medical devices. Some limitations found in the studies are discussed.

## 1. Introduction

Infections caused by microorganisms are a concern for human health. Generally, antibiotics are used for the treatment of bacterial infections, but antibiotic resistance arises and spreads rapidly due to multifactorial causes, such as the natural process of horizontal transference of genes (conjugation, transformation, and transduction) facilitating the spread of antibiotic resistance that can cross several bacterial species mostly associated to an inappropriate use of antibiotics [1]. Virulence factors (such as toxins, enzymes, structural elements, such as flagella, pilli, capsule, etc.) promote disease by either damaging the host or tricking the host immune system, and they need to be neutralized or suppressed for combating bacterial pathogenicity. This is another way to combat those microorganisms with much less possibility to develop bacterial resistance to antibiotics [2].

Essential oils that are obtained from aromatic plants by steam distillation or by mechanical processes from the epicarp of *Citrus*, or “dry” distillation [3] have been described as potential antimicrobial agents due to their capacity for affecting the microbial membrane potential, increasing the membrane permeability, which facilitates the transport of nutrients and ions [4]. The utilization of essential oils, either in liquid or volatile form, as antimicrobial and antivirulence agents were recently reviewed [2,5,6]. Essential oils constituted by dozens or hundreds of compounds can easily undergo degradation reactions (isomerization, polymerization, oxidation, and rearrangements) in the presence of light, oxygen, or moderately high temperatures, leading to the loss or reduction of oil quality. In addition, the intense flavor and aroma may render it difficult to use the essential oils in diverse applications. For these reasons, scientists have developed ways to hide these undesirable characteristics of essential oils and simultaneously enhance their biological properties. Nanotechnology has explored formulations for better using essential oils in diverse fields from the agri-food industry to the pharmaceutical industry [4].

Superparamagnetic iron oxide nanoparticles are constituted by small (<10 nm) synthetic γFe_2_O_3_ (maghemite) or Fe_3_O_4_ (magnetite) particles. They can be coated by organic or inorganic compounds. They also do not possess magnetic moments in the absence of an external magnetic field; nevertheless, when they are submitted to an external magnetic field, they become magnetized. Due to these superparamagnetic properties of nanoparticles, they do not agglomerate and can travel through blood vessels, being manipulated toward a specific body area and/or a target through an external stimulus [7,8,9]. Therefore, the superparamagnetic properties of iron oxide nanoparticles (SPIONs), along with the ability to control their biocompatibility and reduce toxicity, has led to their numerous biomedical applications: drug delivery, hyperthermia for cancer therapy, magnetic resonance imaging (MRI), magnetic particle imaging, magnetic biosensor systems, separation immunoassays, parasite diagnostic assays, nanobridges substances for surgery and wound healing, and cell labeling [10,11,12,13,14,15,16,17,18,19]. The advantage of using SPIONs for biomedical purposes is that they prevent the formation of aggregates as well as guarantee their elimination when they are no longer submitted to a magnetic field [20,21,22,23].

However, those SPIONs must present chemical and colloidal stability, minimum or absent nonspecific interactions, desired pharmacokinetics, an increased efficiency of internalization by target cells, extended blood circulation time, and slow clearance by the reticuloendothelial system, among other properties for being used in treatment and diagnosis. In fact, the use of these materials can lead to some toxicity issues that need to be considered, being the formation of reactive oxygen species (ROS), which causes oxidative stress-mediated responses, which are the main cause of toxicity [24,25]. It is accepted that the production of those ROS results from the leaching of iron ions from the surface or the core of the nanoparticle due to enzymatic activity. The accumulation in different tissues of the body and the characteristics of surface coating of the nanoparticle may also play an important role in the leaching and/or accumulation of iron ions that subsequently could lead to homeostasis imbalance and aberrant cellular responses such as oxidative stress, cytotoxicity, epigenetic events, DNA damages, and inflammatory processes [25]. Therefore, a proper surface coating/functionalization of these nanoparticles is of paramount importance to achieve these attributes [26,27].

## 2. Synthesis of Magnetite Nanoparticles

Different methods have been developed along the past decades aiming at the preparation of magnetite nanoparticles. Nevertheless, there is still room for improvement because if the synthesis of a pure magnetite phase is relatively easy to perform, obtaining magnetite nanoparticles with a desired particle size and acceptable size distribution is a more challenging task. Detailed reviews that include a variety of synthesis strategies of these materials can be found in the literature [28,29,30,31,32,33].

Nanoparticles of magnetite (and maghemite) may be prepared by a large number of synthetic procedures; however, only the foremost used procedures will be described below.

### 2.1. Coprecipitation Methods

The coprecipitation method is the most used and probably the simple and efficient method to prepare magnetite nanoparticles [28,29]. These nanoparticles are usually prepared by mixing Fe(II) and Fe(III) salts with a molar ratio Fe(II)/Fe(III) of 0.5 in an aqueous medium. The precipitation occurs usually at pH in the range 8 to 14, by the addition of a base [34,35,36] (e.g., NaOH, NH_4_OH). The chemical reaction leading to the formation of magnetite may be written as Fe^2+^ + 2 Fe^3+^ + 8 OH^−^ → Fe_3_O_4_ + 4H_2_O.

The iron oxide obtained is recovered by magnetic decantation or centrifugation and treated with concentrated acid or alkaline solutions in order to promote its electrostatic stabilization.

The main advantage of this method is the large quantity of nanoparticles that may be synthetized and some degree of control on the average particle size. Nevertheless, the control of the particle size distribution is rather limited, because the growth of crystals is controlled by kinetic factors [28].

The formation of the nanoparticles follows the usual mechanism of the nucleation and growth of crystals. In this case, a large number of nuclei are formed simultaneously from the homogeneous solution in a process called “burst nucleation” as the species reach a critical supersaturation, which is followed by a stage where a slow growth of the crystals occurs. In order to control the particle size distribution, the nuclei formation must be avoided during this second stage of crystal growth according to the LaMer model [37].

Magnetite does not show a high stability in aqueous environments and may be subjected to oxidation to maghemite (γFe_2_O_3_) in the presence of oxygen. Thus, in order to prevent the oxidation of Fe(II) ions, it is usual to carry out the synthesis process under an inert gas.

Different variations of the classical coprecipitation method have been described in the literature [36]. Among those, the use of Na_2_SO_3_ seems to favor the reaction control in what concerns the oxidation of ferrous ions, avoiding the use of a protective gas (usually nitrogen). In this case, the ferrous ions are not added to the synthesis mixture but instead are generated in situ by the partial reduction of ferric ions with Na_2_SO_3_ [38]. The formation of complexes of ferric ions with SO_3_^2−^ prevents the subsequent oxidation.

Since the size and size distribution of the nanoparticles are of paramount importance in the majority of applications, some strategies have been developed in order to achieve, at least, a partial control over these parameters. The key idea is to cover the nanoparticles with an organic moiety (polymeric or quelating organic anions) during the synthesis process in order to prevent further growth.

### 2.2. Water–Oil Emulsion Methods

The synthesis procedure involving water–oil emulsions is another method that has been used to prepare magnetite nanoparticles for decades. A particularly interesting type of emulsion is the water-in-oil microemulsion (reverse microemulsion), where the aqueous phase is dispersed as microdroplets (typically 1–50 nm in diameter) in a non-polar phase. In this method, the aqueous droplets are stabilized by a thin layer of a surfactant. Each stabilized droplet is a confined environment that can impose kinetic and thermodynamic constraints to chemical reactions involved in the nanoparticles formation and can thus act as a nanoreactor. The size of the reverse micelles can be controlled by varying the water/surfactant ratio, and consequently, this offers a way to control the size and size distribution of the synthetized magnetite nanoparticles. Under stirring, the reverse micelles continuously collide, giving rise to fusions and breakups that make the different precursors present in individual droplets eventually mix and initiate the reaction [39]. Using this method, the formation of the nanoparticles is usually carried out at room temperature but may also be conducted at higher temperature (limited by the boiling point of the organic solvent) [40]. After completion of the reaction, the product may be recovered after adding different solvents, such as methanol or acetone, in order to break up the micelles. A large variety of surfactants such as cetyltrimethylammonium bromide (CTAB), sodium dodecylbenzenesulfonate (NaDBS), Igepal CO-520, Triton-X 100 sodium dodecylsulfate (SDS), polyoxyethylene (Brij30), dodecyltrimethylammonium bromide (DTAB), dodecyltriethylammonium bromide (DEAB), dodecyltributylammonium bromide (DBAB), dimethylene-1,2-*bis*(dodecyldimethylammonium bromide, and Aerosol OT (dioctyl sulfosuccinate) were used to magnetite-based nanoparticles [41,42,43]. In some cases, a co-surfactant is also used.

The method has been shown to be adequate to prepare both pure magnetite nanoparticles and magnetite core–shell structures with different organic and inorganic compounds. It was first described for the synthesis of magnetite sols by Gobe et al. [41] that used microemulsions of water/isooctane and water/cyclohexane with Aerosol OT (dioctyl sulfosuccinate) and hexaoxy–ethylene nonylphenyl ether as surfactants. Since then, a large diversity of nanoparticles has been prepared using the reverse micelles method ranging from metallic or intermetallic materials to quaternary metal oxides.

In what concerns the synthesis of magnetite nanoparticles, this approach has several advantages such as the low temperature of operation, usually 20–70 °C; the reaction time, which typically is in the range of minutes to just a few hours; the good control of the shape and size of the particles; and a narrow size distribution. Several reports have shown the versatility of the microemulsion method, either alone or included into a more complex procedure. For instance, Vidal-Vidal et al. [44] reported the preparation of monodispersed magnetic nanoparticles coated with oleylamine obtained in a water-in-oil emulsion (cyclohexane/Brij-97/aqueous phase). Shen et al. [45] prepared Fe_3_O_4_/carbon core–shell nanoparticles using aryl sulfonyl acetic acid and glucose, involving a thermal decarboxylation to give Fe_3_O_4_ from iron aryl sulfonyl acetate with a reverse microemulsion process and a solvothermal reaction of glucose to prepare carbon in nanoscale.

### 2.3. High-Temperature Methods

Different strategies for the synthesis of magnetic nanoparticles, involving high temperature, have also been successfully developed. These high-temperature methods that include hydrothermal, solvothermal, and thermal decomposition usually require the synthesis mixture to be placed into a sealed autoclave and heated at temperatures well above the boiling point of the solvent and sometimes even at temperatures above its critical temperature. Variations of this method may include the use of a continuous system involving a counter-current flow reactor [46].

When compared with the coprecipitation method, high-temperature procedures give rise to a better control of the size, size distribution, and shape of magnetic nanoparticles [30,47]. As in the coprecipitation method, different additives (such as oleic acid, sucrose, poly(acrylic acid), ionic liquids, oleylamine, poly(ethylene glycol), 1-octadecene, amino acids, citric acid, etc.) have been used in order promote the stabilization of the nanoparticles and prevent particle aggregation and in some cases to promote a one-step functionalization [36,39,48,49,50,51,52].

Magnetic nanoparticles can be prepared in different ways involving the oxidation, reduction, or coprecipitation of iron precursors. Ge and co-workers [53] have shown that magnetite nanoparticles with different particle sizes can be prepared from FeCl_2_ and ammonia through a hydrothermal or solvothermal treatment, changing the reaction conditions. Here, air is used before and during the thermal treatment to promote the oxidation of Fe(II) to Fe(III). Tian et al. [54] used Fe(acac)_3_ (iron(III) (acethylacetonate)) as an iron source, *n*-octylamine as a reductant, and *n*-octanol as a solvent to prepare ultrasmall monodisperse magnetite nanoparticles. By varying the composition of the synthesis mixture, namely the volumes of *n*-octanol and *n*-octylamine, monodisperse nanoparticles with sizes of 1, 4, 5, and 6 nm could be obtained. A coprecipitation of FeCl_2_ and FeCl_3_ at 70 °C followed by a hydrothermal treatment at 250 °C was the method followed by T.G. Daou et al. [55] to prepare magnetite nanoparticles with an average size of 39 nm with good monodispersivity. To prevent oxidation, argon was used during the whole process.

The scope of hydrothermal and solvothermal approaches has been expanded; one the main targets today is the preparation of magnetic nanoparticles with unusual morphologies. The works, among many others, of Wan et al. [56], Thomas et al. [46] or Liang et al. [57] are examples of the potential exhibited by these methods, where mesoporous nanocrystals, particle aggregates with a flower-shape structure, and well-faceted single crystals were obtained, respectively.

The thermal decomposition of iron precursors in organic solvents, using organic fatty acids or amines as stabilizers, is another strategy that allows the preparation of size-controlled and monodisperse magnetic nanoparticles with high crystallinity [58], although a high temperature is usually required. These methods may be implemented in open or closed containers depending on the type of iron precursor, stabilizer of functionalizing agent added, and textural properties expected.

A usual procedure involves the preparation of an iron–oleate complex that is subsequently heated at high temperature in the presence of oleic acid in a high-boiling point solvent as described by Park [59] and Li [60]. In this approach, the operational temperature depends on the choice of the solvent. By a similar procedure Patsula [61] and co-workers described the preparation of superparamagnetic Fe_3_O_4_ nanoparticles via the thermal decomposition of a Fe(III)-glucuronate complex synthetized in a previous step. Fe(acac)_3_ is another widely used iron precursor that allows a simple implementation of the method [62,63,64,65].

High-temperature methods are well suited for the control of the particle size, since the crystal growth is mainly achieved at the high-temperature stage. Consequently, the crystal growth can be easily stopped by a quick decrease of the reaction temperature. Other parameters, such as the Fe/oleic acid ratio, the solvent used, and the addition of sodium oleate have also a direct influence on the particle shape and particle size [66,67].

The thermal decomposition of iron precursors can also be accomplished in solventless conditions as shown by Asuha et al. [68], who prepared magnetite nanocrystals through a direct thermal decomposition in a closed container of an iron–urea complex ([Fe(CON_2_H_4_)_6_](NO_3_)_3_) [68]. Using this procedure, the size of the nanocrystals is dependent on the temperature of decomposition. Urea, one of the products of the thermal decomposition of the initial complex, seems to play a key role in the formation of magnetite by the partial reduction of the F^3+^ ions.

### 2.4. Sol–Gel Methods

The sol–gel method, another class of synthesis procedures, is based on the hydrolysis and condensation reactions of metallic precursors in solution, in such conditions where a sol of nanometric particles is achieved. Further condensation and polymerization reactions give rise to a three-dimensional network of either discrete particles or network polymers, which are usually known as wet gel. Usually, these reactions are performed at low temperature; thus, further treatments at higher temperature may be needed to obtain the final structure.

Magnetite nanoparticles have been prepared though this approach and, in a similar way to that found for other type of nanoparticles, the structure and properties of the gel are influenced by the solvent, temperature, nature, concentration of the salt precursors employed, pH, and agitation [28]. The most used precursors for the synthesis of magnetic nanoparticles are iron alkoxides and iron salts (such as chlorides, nitrates, and acetates), which undergo various forms of hydrolysis and polycondensation reactions in acidic or alkaline conditions.

Through this method, it is possible to obtain materials with a predetermined structure according to the experimental conditions, with good control of the particle size and homogeneity of the reaction products [69]. The possibility to embed molecules, which maintain their stability and properties within the sol–gel matrix, makes this method particularly suited for the preparation of core–shell nanoparticles and organic–inorganic composites involving magnetic oxides. For instance, Hasanpour et al. [70] prepared magnetite nanoparticles with an average size of 10 nm starting from ferric nitrate nonahydrate and ethylene glycol. The sol was obtained at 40 °C, under stirring for 2 h, and the gel was obtained at 80 °C; a subsequent drying was performed at 150 °C for 20 h, and the annealing was done at different temperatures (350–650 °C) for a further 2 h. The annealing temperature plays an important role in the process, because it induces different types of crystallinity, size, and also magnetic properties of the particles. The authors have shown that this synthesis route can be also used for the “one-pot synthesis” of Fe_3_O_4_/ZnO nanocomposite materials. FexOy–SiO_2_ composites have been widely prepared by procedures that involve a sol–gel step as shown by Raileanu et al. [71], who prepared this type of composites using alkoxide and aqueous routes and different precursors of silica (tetramethoxysilane, methyltriethoxysilane, colloidal silica solution, etc.) or Chae et al. [72], who prepared core–shell Fe_3_O_4_@SiO_2_ nanoparticles by a two-step method: Fe_3_O_4_ nanoparticles were prepared by a solvothermal method, and in the second step, the Fe_3_O_4_ nanoparticles were coated with SiO_2_ formed through the hydrolyzation of tetraethyl orthosilicate.

Less conventional sol–gel procedures were also adopted by different authors to prepare magnetic nanoparticles. For instance, Lemine et al. [73] synthetized magnetite nanoparticles with an average size around 8 nm using the modified sol–gel method under supercritical conditions of ethyl alcohol, and Sciancalepore et al. [74] prepared monocrystalline magnetite particles with a size ranging from 4 to 8 nm using a microwave-assisted non-hydrolitic sol–gel synthesis route.

### 2.5. Other Methods

Numerous other methods have been used for the preparation of magnetic nanoparticles or magnetic nanoparticles containing composites; these include polyol synthesis, microwave-assisted synthesis, sonolysis, flow injection synthesis, electrochemical methods, aerosol/vapor methods, and biosynthesis. Some of these methods can be understood as improvements of one of the above-described methods. For example, heating under microwave radiation can significantly reduce processing time and energy cost due to the very fast heating of the synthesis mixture resulting from the strong agitation of the reorientation of the molecules in phase with the electrical field excitation [36]. Recently, Kubrakova et al. [75] have used microwave radiation (80 °C and 8–15 min irradiation time) to promote the formation of magnetite by coprecipitation. Under this procedure, the authors obtained both naked and surface-modified magnetite with oleic and mercaptopropionic acids and cetyltrimethylammonium bromide. Very high-temperature hot spots can be generated by the use of ultrasound. These hot spots result from the rapid collapse of ultrasonic-generated cavities and may be used to prepare magnetic nanoparticle by the decomposition of iron precursors as shown by Abu Mukh-Qasem [76] or coprecipitation as reported by Nan Wang [77].

The polyol method comprises a polyol as solvent where an iron precursor is suspended. The mixture is stirred and heated at a given temperature that is limited by the boiling point of the polyol used. When compared with the sol–gel method, this approach makes use of a reduction reaction of the iron precursor instead of the oxidation that occurs in the sol–gel process. This is the reason why this method is also called an inversed sol–gel method. Polyols, such as ethyleneglycol or triethylene glycol, may dissolve inorganic compounds due to their high dielectric constants and may offer a wide range of working temperatures due to the relatively high boiling points. In this process, polyols also serve as reducing agents and stabilizers that prevent particle aggregation [28,36].

Flow injection methods consist of the continuous or segmented mixing of reagents under a laminar flow regime in a capillary reactor. These methods are in fact modified coprecipitation methods but exhibit properties such as high reproducibility, high mixing homogeneity, and an opportunity for a precise external control of the process [28,36]. Electrochemical methods may also present some advantages over other synthesis methods, namely the high purity of the product and the possibility of controlling the particle size by the adjustment of the current or potential applied to the system [78,79].

Spray pyrolysis and related methods, examples of aerosol technologies for the synthesis of magnetic nanoparticles, are continuous chemical processes that allow a high rate of production. Spray pyrolysis consists in the spraying of a solution of ferric salts and a reducing agent in organic solvent into a series of reactors, where the aerosol solute condenses, the solvent evaporates, and a pyrolysis reaction takes place (usually at temperatures above 500 °C). The resulting dried residue consists of particles whose size depends upon the initial size and size distribution of the original droplets [28,36]. This particular method shows many advantages in the synthesis of pure magnetic nanoparticles and magnetic nanoparticles-containing composites as it is a continous flow process, where the nanoparticles are synthetised in a sigle step with lower costs than other processes. The method is easy to implement and exhibits a high chemical flexibility that allows the preparation of particles of different materials with different compositions [80,81,82]. Several variations of this method have been described in recent years. Examples of these are the use of ultrasounds for dispersing the precursor solution into droplets (ultrasound spray pyrolysis), as reported by Peter Majeric et al. [83] in the preparation of maghemite nanoparticles (260–390 nm) decorated with Au nanoparticles (24–67 nm), the spraying of aqueous metal solutions directly into a flame (flame spray pyrolysis) used by Reto Strobel and Sotiris E. Pratsinis [84] to prepare maghemite, magnetite, and wustite nanoparticles, or the use of a laser beam to promote the pyrolysis of iron precursors as repoted by Sabino Veintemillas-Verdaguer et al. [85].

All the methods described above present advantages and drawbacks in terms of the simplicity and control of the shape, size, and size distribution of the nanoparticles. When choosing a synthesis method, the requirements of final application in terms of amount, size, shape, size distribution, surface properties, and magnetic properties of the nanoparticles have to be considered. Table 1 summarizes some general trends observed in representative methods.

## 3. Stabilization and Surface Modification

The stabilization of the magnetic nanoparticles as a magnetic colloidal suspension is a central issue that has to be considered in any subsequent application [28,30,36]. In this range of particle size, it is well known that the particles tend to aggregate in order to reduce the surface energy. On the other hand, the chemical stability, namely that of the magnetic nanoparticles, has to be assured since the easily oxidation of the iron atoms in the magnetite structure causes changes in the magnetic properties. Finally, a surface coating of the nanoparticles with appropriate molecules becomes a necessity if a medical application is envisaged.

The iron atoms in the particle surface act as Lewis acids and therefore may coordinate with atoms of molecules that can donate a pair of electrons. In aqueous solutions, water coordinates with Fe easily dissociate, giving rise to a hydroxyl-functionalized surface. The hydroxyl groups are amphoteric, and the surface of the nanoparticles may be positive or negative depending on the pH of the solution.

**Figure 1:**
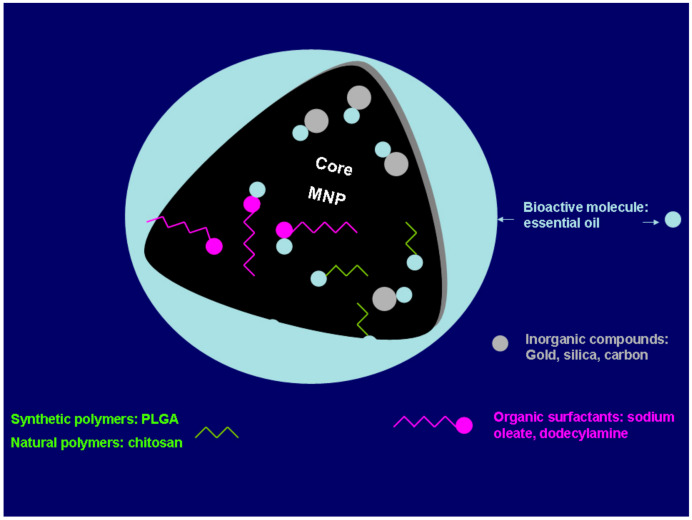
Schematic representation of the different strategies available to obtain water-soluble and chemically active nanoparticles. The final ligands on the nanoparticles can be either small molecules or polymers. (MNP—magnetic nanoparticles). Adapted from Wallyn et al. [86].

All the synthesis methods described above take into consideration at least the aggregation issue and make use of additives to prevent this occurrence, such as citric or oleic acids. Nevertheless, in most of the cases, the methods used to prepare monodisperse nanoparticles with high crystallinity use hydrophobic surfactants to cover the particles surface, which makes them insoluble in water. A direct medical or biological application of such nanoparticles is not usually possible, and surface modification is required in order to obtain water-dispersible and chemically stable biocompatible magnetic materials. This surface modification may either be in situ or post synthetic. The most common strategies to fulfill these requirements involve the formation of core–shell structures where the surface of the magnetic nanoparticles is modified with surfactant micelles, bi-functional ligands, polymeric ligands, or silica (Figure 1) (in this case, the use of silica may be an intermediate step for subsequent functionalization).

These modifications can be attained either by ligand exchange, organic coating, or inorganic coating [30,36,87,88]. Those modifications can be inorganic metals (gold); inorganic oxides (carbon, silica); synthetic polymers [poly(ethylene-co-vinyl acetate), poly(vinylpyrrolidone) (PVP), poly(lactic-coglycolic acid) (PLGA), polyethylene glycol (PEG) and polyvinyl alcohol (PVA)]; natural polymers (dextran, pullulan, gelatine, chitosan); and organic surfactants (sodium oleate, oleic acid, dodecylamine), among other examples [27,89,90,91]. According to Sarkar et al. [92], the surface modification of magnetite nanoparticles can also be done by annealing the nanoparticles with eucalyptus leaves at relative high temperatures (500–800 °C), without losing the superparamagnetic character and making them hydrophilic and biocompatible, which are important factors for biomedical applications. Other authors [93] also showed the possible surface modification using nutmeg essential oil. This green synthesis of magnetite nanoparticles improved the saturation magnetization.

## 4. Superparamagnetic Iron Oxide Nanoparticles for Extracting Essential Oils versus Essential Oils for Improving or Producing Superparamagnetic Iron Oxide Nanoparticles

The extraction of essential oils is made by steam distillation or hydrodistillation with the exception of those extracted from the epicarp of *Citrus* sp. Those procedures are time consuming; some compounds cannot be extracted, and other ones can undergo degradation reactions. Other classical and conventional methods have also been used for extracting the volatile fraction of aromatic plants such as solvent extraction, enfleurage, or hydrodiffusion, all of them with advantages and disadvantages. Other innovative extraction methods have been developed in order to minimize some disadvantages of those procedures, such as supercritical fluid extraction, subcritical fluid extraction, instantaneous controlled pressure drop process, microwave-assisted extractions, and ultrasound-assisted extraction; all of these methods have been reviewed recently [94,95].

According to the review made by Mejri et al. [95], microwave-assisted extractions include diverse techniques, such as microwave-assisted ionic liquids treatment followed by hydrodistillation, microwave-assisted solvent extraction, compressed air microwave distillation, vacuum microwave hydrodistillation, microwave-accelerated steam distillation, microwave hydrodiffusion, and gravity and solvent-free microwave extraction. Sometimes, it is possible to couple microwave distillation extraction with separation and identification techniques—for example, head space/solid phase microextraction (HS/SPME), as performed by Ye and Zheng [96]. According to these authors, the extraction of the volatile fraction from dried aromatic plants with little moisture makes the extraction by microwave method complicated, owing to the little amount of water to absorb microwave energy. For this reason, the addition of magnetite materials, which are good absorbers of microwave radiation, was a way to solve the problem. The authors [96] used amine-functionalized magnetite nanoparticles as a microwave absorption solid medium for extracting the volatile fraction of *Perilla frutescens* (L.), which was followed by the identification and quantification of the compounds by HS/SPME. The same approach was also performed by Hashemi-Moghaddam [97], who compared the microwave-assisted hydrodistillation using amine-functionalized magnetite nanoparticles with the classical hydrosdistillation method for extracting the essential oil of the hulls of *Pistacia vera* L. The presence of amine-functionalized magnetite nanoparticles increased the oil yield, and more compounds were possible to be extracted, although the main components (α-pinene, α-terpinolene, myrcene, pinocarvone and camphene) did not alter.

Beyond magnetite nanoparticles being used for extracting essential oils, essential oils can be added themselves to magnetite nanoparticles for enhancing the colloidal stability of the manufactured magnetic fluid sample through innovative extraction methods. These studies have been particularly developed by several authors [98,99], using copaiba Amazonian natural products, such as copaiba oil (*Copaifera Langsdorffii* Desf.) oil, and the nanoparticles were obtained by coprecipitation in alkaline medium using a mixture of aqueous solution containing ferrous chloride and ferric chloride. They reported differences of Mössbauer parameters for iron oxide nanoparticles as prepared and dispersed in copaiba oil. According to the authors, these differences could be attributed to the interactions of polar molecules of copaiba oil with Fe^3+^ at the nanoparticles surface [99], although Santos et al. [100], by photoacoustic spectroscopy, concluded that copaiba molecules could attach to the nanoparticle surface through hydrogen bonding with a natural polyoxyhydroxi layer. A nanofluid was also obtained by Gaspar et al. [101] after the incorporation of superparamagnetic iron oxide nanoparticles, obtained by thermal decomposition procedures, into the Amazonian copaiba and andiroba (*Carapa guianensis* species) oils, always preserving the superparamagnetic behavior but with low saturation magnetization. However, it is important to stress that the chemical composition of both oils is particularly different. Whereas copaiba oil is an oleoresin mainly constituted by diterpenes and sesquiterpenes (volatile compounds), andiroba oil is mainly constituted by triacyglycerols composed by palmitic, oleic, and linoleic acids [101,102].

Still from Amazonian origin, the essential oil of *Croton cajucara* leaves was used by several authors [103,104] during the oleic acid-coated magnetic nanoparticles synthesis, by the thermal decomposition method; these authors studied whether the essential oil could constitute a second surface layer on the oleic acid-coated magnetic nanoparticles. The authors reported that the bilayer-coated magnetic nanoparticles with *C. cajucara* oil were successfully produced, and their structural and morphological characterizations were performed through several techniques (X-ray diffraction, transmission electron microscopy, and high-resolution transmission electron microscopy, thermogravimetric analysis, and magnetic measurements). In addition, the authors also verified that the double-layer (oleic acid plus essential oil) coating of the magnetite nanoparticles noticeably quenches the particle–particle interaction originating a superparamagnetic-like behavior [103,104]. Furthermore, the authors [103] found, by thermogravimetric analysis, that the release of the essential oil occurred at approximately 50 °C, whereas the oleic acid was released only above approximately 380 °C. According to the authors [103], the combination of the improved magnetic properties due to the double layer (oleic acid plus essential oil) coating of the magnetite nanoparticles which quenches the particle–particle interaction along with the detachment of essential oil at approximately 50 °C, keeping always stable the oleic acid coating, could render these double-layer magnetite nanoparticles candidates for site-delivering essential oils for targets to treat specific diseases.

The essential oils obtained from *Syzygium aromaticum* (clove) or *Cinnamomum* sp. were used for producing magnetite particles by the sol–gel method combined with autoignition. In this procedure, nitrates are used as oxidants, and organic molecules (glycine, citric acid, or urea) are used as fuels but with the disadvantage of lacking antioxidant attributes. For this reason, Ben-Arfa et al. [105] used cinnamom and clove oils as fuels and antioxidants for preserving the magnetic phases during subsequent heating of the production, which means that H_2_/N_2_ or argon gases were not required during the heat stabilization process. The samples were stabilized in air at 550 °C. The magnetic crystalline phase was mostly maghemite (γFe_2_O_3_) rather than magnetite (Fe_3_O_4_), but with identical magnetic properties. However, clove oil produced magnetic iron oxide particles more susceptible to undergoing thermally induced oxidation upon heating in air, and cinnamon possessed stronger anti-oxidative resistance [105]. The magnetic iron oxide particles were under clusters of agglomerates in the order of 3–30 μm with non-uniform shapes and sizes, in which the individual particles sizes were of hundreds of nm [105].

## 5. Superparamagnetic Iron Oxide Nanoparticles Applications

Iron, cobalt, and nickel, generally under alloys (e.g., FeCo, FePt, CoPt, and FePd), oxides (e.g., Fe_3_O_4_, Fe_2_O_3_, and MnO), and ferrite nanoparticles (MnFe_2_O_4_, NiFe_2_O_4_, and ZnFe_2_O_4_) or composites (e.g., Fe_3_O_4_–linoleic acid) have been reported for diverse applications [106,107], although Fe_3_O_4_ and γFe_2_O_3_ nanoparticles are the mostly utilized iron oxide nanoparticles because of their superparamagnetism, biocompatibility, and lower toxicity [108,109]. The superparamagnetic characteristic of these nanoparticles means that in the absence of an external magnetic field, they lose magnetic momentum, becoming non-magnetic, but a mean magnetic momentum appears if an external field is applied. These properties make these superparamagnetic nanoparticles interesting for carrying drugs to the specific target sites at relative high levels because they are guided to those target sites through an external magnetic field or receptor targeting. This behavior reduces substantially the adverse and toxic effects in other parts of the organism [7,8,9].

However, the magnetism of SPIONs can be hampered due to possible agglomeration and the risk of oxidation by air exposure; therefore, coating these nanoparticles is necessary, and this is usually carried out during and post synthesis with diverse non-degrading and nontoxic materials, including polymers such as starch and dextran, chitosan, carboxymethylcelulose, proteins (albumin), Arg–Gly–Asp (RGD) peptides, and lipids (fatty acids and their esters), and synthetic polymers, such as polyethylene glycol, polyvinyl alcohol, poly (acrylic acid) are only some examples [110] or even the encapsulation of SPIONs in liposomes, thermoresponsive hydrogels, and dendrimers [111,112,113]. These coatings not only stabilize the nanoparticles and assist in their dispersion but also may have a role in the disease therapy after functionalization with drugs for delivering them on the target. They may also improve the pharmacokinetic properties of drugs [110,111], increase magnetic resonance signals, or even make the nanoparticles theranostics; that is, they are able to work as diagnostics and include treatment by a single intervention [113,114]. The utilization of polyethylene glycol is generally used for inhibiting phagocytosis by the reticuloendothelial system, improving the half-life of SPIONs in blood circulation and simultaneously promoting the enhanced permeability and retention effect in vivo [114].

It is predicted that most nanoparticles can accumulate much better within cancer cells, due to their poorer lymphatic drainage, compared to normal cells, and therefore, there is an enhanced permeability and retention effect [115]. However, there is always heterogeneous behavior, since tumors are heterogeneous by nature (diverse tumor types of the same origin, tumors at different locations in the same patient, and even at different stages of the same tumor during its development). In addition, within a tumor, there are variations in the thickness and density of the extracellular matrix, irregular blood flow distribution, and unequal vessel permeability [116]. These limitations have led to the development of new strategies for overcoming the enhanced permeability and retention effect alone in cancer nanomedicine [116].

Other applications have been done for superparamagnetic iron oxide nanoparticles: hyperthermia for cancer therapy, magnetic resonance imaging (MRI), magnetic particle imaging, magnetic biosensor systems, separation immunoassays, parasite diagnostic assays, nanobridges substances for surgery and wound healing, modulation of the regenerative effects of mesenchymal stem cells [117], and cell labeling [10,11,12,13,14,15,16,17,18,19]. However, geometric shapes and/or the size, surface coating, and charge of the magnetic nanoparticles have an important role in their efficacy [118].

There are diverse geometric forms of SPIONs depending on the synthesis: spherical, cubic, hexagonal, rod-like, octagonal, nanoworm, and octopod (star-shaped) SPIONs [118]. Nevertheless, 20 nm-size single-domain cubic iron oxide particles are better for hyperthermia applications than spherical particles; 30 nm-size octopod SPIONs are better than spherical ones for MRI; or even nanoworms are better for targeting tumors in in vitro assays and for accumulation in tumors (in vivo) than simple spherical SPIONs [116,119,120].

SPIONs having sizes between 5 and 10 nm are better for treatments needing slow drug release; however, these sizes may contribute to a capillary blockage because they can be retained in the blood circulation for longer periods. In addition, these size ranges can easily enter into the cell nucleus, inducing DNA damage (Suciu et al., 2020). Nevertheless, there are reports considering that these very small sizes are a good characteristic of SPIONs, particularly for gene therapy [121]. In spite of those drawbacks, 5 to 15 nm SPIONs are presently approved and used for human medical applications, such as MRI (Suciu et al., 2020). Particles with sizes higher than 200 nm become concentrated in the spleen or are taken up by phagocytic cells, leading to low plasma concentrations (Nguyen et al., 2016).

Polyvinylpyrrolidone-coated SPIONs, at concentrations ranging from 10 to 100 μg/mL, and for up to three days, promoted the proliferation of human breast cancer BT-474 cell line. This behavior was not observed when the concentration of SPIONs were higher than 200 μg/mL or when the coating was dextran. For higher concentrations, polyvinylpyrrolidone-coated SPIONs were toxic to these cancer cells, as desired, and dextran-coated SPIONs, at concentrations lower than 200 μg/mL, were toxic for the same cancer lines after 48 h of exposition [122].

Cell surfaces generally have a negatively charged outer layer; therefore, positively charged particles tend to attach to the cell surfaces, which may become toxic to these cells. Positively charged polyethyleneimine–Fe_2_O_3_–NPs (PEI-NPs) were toxic in utero of mice exposed to these nanoparticles, whereas negatively charged poly(acrylic acid)–Fe_2_O_3_–NPs (PAA-NPs) only induced mild toxicity [123].

Magnetic particle imaging (MPI) measures and maps the concentration of SPIONs over a spatial position. In magnetic particle spectroscopy (MPS), sinusoidal magnetic fields are applied to periodically magnetize SPIONs; then, the dynamic magnetic responses of SPIONs, which contain unique higher odd harmonics, are harvested by a pair of pick-up coils [124]. These harmonics are useful metrics for characterizing the magnetic nanoparticle ferrofluids. This principle was used for quantitatively detecting H1N1 nucleoprotein of Influenza A Virus (IAV) subtype H1N1. The authors [125] anchored IgG polyclonal antibodies onto SPIONs surfaces. The H1N1 nucleoprotein, which hosts multiple different epitopes for these IgG polyclonal antibodies, are able to cross-link between SPIONs (IgG from the particle surface) and H1N1 nucleoproteins, forming different degrees of SPIONs clusters, depending on the number/concentration of H1N1 nucleoprotein molecules in the magnetic nanoparticle ferrofluid. When these ferrofluid samples are subjected to external oscillating magnetic fields, significant changes in the macroscopic magnetic responses of magnetic nanoparticles occur, which are detected by the MPS system. According to the authors (Wu et al., 2020), this approach was able to detect concentrations in the range of hundreds of pmole of H1N1 nucleoprotein. The method was also rapid (results provided in 10 s), needing only non-technicians with minimum technical training. This method shows some importance to be improved for several reasons; for example, according to the World Health Organization (WHO), influenza viruses, responsible for seasonal epidemics of acute respiratory illness known as influenza or flu, are responsible for 250,000 to 500,000 deaths annually [124]. In this review article, the authors consider that this approach must be explored for the detection of SARS-CoV-2, since current diagnostic tests are based on real-time reverse transcription-polymerase chain reaction (RT-PCR) that requires 48 h for having a result, expensive equipment, and trained technicians, and there have been many global deaths. At the end of August 2020, 8 months after the first notifications of atypical pneumonia in the Hospital of Wuhan, China, more than 840,000 of deaths were registered due to the SARS-CoV-2 (https://coronavirus.jhu.edu/map.html). Many other MPS-based applications have been reported, which were compiled by [124].

Several components may constitute the functionalized outer coating depending particularly on the intended application. For example, diverse biological molecules [insulin, nerve growth factor (NGF), ceruloplasmin, elastin, Tat-peptide, folic acid, transferring, lactoferrin, transforming growth factor α (TGF-α), gluthatione (GSH), methotrexate, antibiotics, essential oils, and ascorbic acid, among many others] can be bound to the coating polymer in order to direct them to specific targets [11,12,14,21,91,126,127,128,129]. In the cases where it is intended to deliver drugs in specific targets or to make easier the degradation of the nanostructure, the non-specific physical sorption is preferred between the magnetite nanoparticles and the surface-modifying molecule, although non-covalent or covalent bindings may also occur [130].

Superparamagnetic iron oxide nanoparticles may also be applied for removing environmental pollutants that are either organic (dyes, hydrophobic organic compounds) [131,132] or inorganic (arsenic) [133,134] as high-surface-area supports in catalytic transformations [50,135]; as pigments and inks; as anode material for lithium ion batteries [136,137]; in the petroleum industry [138]; or for promoting the osteoblasts proliferation if the iron oxide nanoparticles had a layer of calcium phosphate. In the same study, the authors found that magnetite and maghemite nanoparticles had the capacity for inhibiting the growth of *Staphyloccocus sureus* [139]. Magnetite nanoparticles were also able to inhibit the growth of planktonic cells of *Sacharomyces cerevisiae* yeast but also the biofilm development on glass coverslips, as determined by a microtiter method and the biofilm architecture examined by inverted microscopy and confocal laser microscopy scanning [140]. Later on, Darwish et al. [141], after synthesizing magnetite nanoparticles by coprecipitation and coating them with three functional layers (oleic acid, polyethyleneimine, and polyethyleneimine-methyl cellulose), reported that they were able to prevent the growth of *Staphylococcus aureus* and *Escherichia coli*, particularly those coated with polyethyleneimine-methyl cellulose, and they also inhibited the formation of biofilms of *S. aureus*. All of them were effective for heat generation in alternating magnetic fields, whereby these nanoparticles had self-heating action and antibacterial effects.

The functionalization of the magnetite nanoparticles with antibiotics has been performed with the objective of increasing the molecule activity. For example, functionalized magnetite nanostructures with amoxicilline showed activity against both the Gram-positive *S. aureus* and the Gram-negative *E. coli*, reducing significantly the minimum inhibitory concentration (MIC) of the antibiotic. This result may mean that the therapeutic dose of amoxicillin can be reduced if under functionalized magnetite nanostructures [142]. The same research team [126] also reported a prolonged anti-biofilm activity against *S. aureus* and *Pseudomonas aeruginosa* in the presence of magnetite nanoparticles stabilized by sodium lauryl sulfate and coated with cefotaxime and cefrom antibiotics.

Essential oils (EOs) have been described as possessing antimicrobial activity; several studies have been done with functionalized magnetite nanoparticles with essential oils in order to improve their biological activities. The functionalization of magnetite nanoparticles with EOs with antimicrobial activity will be reviewed in the next section.

## 6. Superparamagnetic Iron Oxide Nanoparticles Supplemented with Essential Oils for Combating Microbial Biofilms

The major concern in the infectious processes is the formation of biofilms by pathogenic microorganisms, which after adherence to surfaces exhibit increasing antibiotic resistance by around 1000-folds [143,144]. The utilization of medical devices, such as catheters, cardiac pacemakers, joint prosthesis, prosthetic heart valves, contact lenses, or implants, among other medical components, are responsible for at least 50% of the nosocomial infections [145]. At least two strategies can be adopted for avoiding the formation of biofilms, such as interference with bacterial adherence through direct blockage of their surface receptors or the utilization of compounds with anti-adherence properties.

Chelating agents, peptide antibiotics, lantibiotics, synthetic chemical compounds, and herbal active compounds are some examples of compounds that have been tested [145,146]. Within the plant-origin compounds, EOs were proved to be able to prevent the formation of biofilms [147,148,149]. The ability of EOs to inhibit microbial adherence and further biofilm development is associated by their capacity to disrupt the quorum sensing system (QS) [148,150,151,152,153]. QS is a largely distributed cell–cell communication process that has been identified not only in bacteria, but also in fungi, protozoa, and recently in bacteriophages [154,155,156,157,158]. An extensive number of physiological functions, including biofilm formation, the expression of virulence factors, conjugation, and competence are regulated by QS through a very finely tuned system of production, secretion, sensing, and DNA interaction of particular molecules that are designated by autoinducers [159]. Since these natural compounds may act as an anti-biofilm, the conjugation of magnetite nanoparticles and natural compounds could be an interesting approach to combat the adherence and biofilm formation.

The capacity of magnetite/oleic acid nanoparticles functionalized with essential oils (*Eugenia caryophyllata* or *Rosmarinus officinalis*), obtained by a precipitation method under microwave condition, was able to inhibit the adherence and fungal biofilm development on functionalized catheter samples [129,160]. The chemical composition of both essential oils was different; whereas the main components of *E. caryophyllata* were eugenol and α-caryophyllene, *R. officinalis* oil was predominantly constituted by 1,8-cineole, α-pinene, camphor, and caryophyllene. The magnetite/oleic acid nanoparticles functionalized with *E. caryophyllata* oil strongly inhibited the adherence of diverse *Candida* clinical species (*C. albicans*, *C. tropicalis*, *C. krusei*, and *C. glabrata*) on catheter surface devices and, consequently, prevented the biofilm formation [160]. *R. officinalis* essential oil-coated nanoparticles also strongly inhibited the formation of biofilm of the clinical *C. albicans* and *C. tropicalis* strains on catheter surfaces observed through confocal laser scanning microscopy and viable cell counts [129].

*Candida* species can be a problem in prosthetic device-related infections; also, *P. aeruginosa* and *S. aureus* are a concern in wound infections. Anghel et al. [146] studied the antimicrobial capacity of magnetite-based nanostructures obtained by wet chemical precipitation using sodium palmitate as surfactant and functionalized with some volatiles (eugenol or limonene). They also studied their anti-adherence properties in order to evaluate the anti-biofilm formation by those microorganisms on a cotton-based material generally used for covering infected wounds. This approach had also the capacity of stabilizing and controlling the release of the volatiles that under the free form would volatilize and would be no more present after some hours of application on the cotton surface. Those authors [146] showed that nanofluid coating containing limonene affected both the initial stage of biofilm formation and its development after 1, 2, or 3 days on coated textile materials for *P. aeruginosa* and *S. aureus*; nevertheless, the effect of nanofluid coating containing eugenol was more pronounced on adherence and initial biofilm formation in comparison with the limonene-based one for *P. aeruginosa*. These results proved that the functionalized textile material cumulated the anti-adherence ability of magnetite nanoparticles with the antimicrobial activity of eugenol and limonene against *P. aeruginosa* and *S. aureus*, which were frequently detected in cutaneous wound infections, without their release by volatilization, [146]. The same approach was also performed by Pentru et al. [161] but replacing limonene and eugenol by carvone against the growth of *C. tropicalis* strains and in the inhibition of biofilm formation on the traditional cotton wound dressing, which is used for covering infected wounds. The authors [161] found that the functionalized magnetite nanoparticles/stearic acid with carvone applied on the cotton surface acted in the initial steps of biofilm formation (adherence and micro-colonies forming) as well as in the development of mature biofilms (activity checked after 3 days). Anghel et al. [162] found that the *Satureja hortensis* essential oil, mainly constituted by carvacrol and γ-terpinene, when associated with functionalized magnetite nanoparticles/oleic acid also inhibited not only the adherence of *C. albicans* on the cotton wound dressing but also the development of biofilm.

Vanilla, patchouli, and ylang-ylang essential oils have been reported as being effective against several microorganisms [163]; nevertheless, their intrinsic volatilities make them difficult to use as antimicrobial agents. In previous studies and as aforementioned, magnetite nanoparticles with adequate surfactants (oleic acid, sodium palmitate) associated with other essential oils with unknown chemical composition showed to be able to inhibit the adherence of clinical yeasts strains and as well Gram-positive and Gram-negative bacteria or their biofilm formation not only on cotton wound dressing but also on catheter surfaces [163], demonstrating the sucessful impact of novel nanobiosystems constituted by magnetite nanoparticles on the control of microbial growth and their community mode of life.

Iron oxide nanoparticles functionalized with patchouli essential oil, with unknown chemical composition, were used to develop a biocompatible coating for wound dressings using the coprecipitation of a precursor in an alkaline solution of patchouli oil [164]. These functionalized magnetite nanoparticles showed good anti-staphylococcal biofilm (*S. aureus* ATCC 25923) activity after 24 and 48 h, but after 72 h, its anti-biofilm action reduced markedly in contrast with that previous reported for magnetite nanoparticles functionalized with poly lactic acid/chitosan polymers and the essential oil isolated from *Melissa officinalis*, according to the study of Grumezescu et al. [165]. In this case, and in order to test the anti-biofilm properties of the nanoparticles, they were distrubuted on wells of a 6-well microplate, and the bacterial suspension was added and its viability was determined over 72 h. The authors observed that throughout this time interval, the biofilms formed on the nanomodified coating was significantly lower (<3 times) in comparison with biofilms established on uncoated surfaces, evidencing the advantage of using natural bioactive compounds that can be useful to combat resistant forms of bacterial pathogens. The method of Matrix-Assisted Pulsed Laser Evaporation (MAPLE) was used to produce the bioactive film containing magnetite nanoparticles and the *Melissa* essential oil that was mainly constituted by 28% limonene, 31% β-citronellal, 15% β-citral, 9% α-citral, and 8% caryophyllene. In this experiment, the biological activity extends over a longer period of time than that previously reported [124], leading these authors to suggest replacing the coated wound dressing every day.

Iordache et al. [166] used an identical approach to the one described in the study of Grumezescu et al. [165], but instead of the essential oil of *M. officinalis*, they used the essential oil of *Cinnamomi aetheroleum* in the functionalization of the bioactive thin film constituted by poly(lactic-co-glycolic) acid/chitosan microsphere coatings containing essential oil. The authors observed that the biofilm formed by *S. aureus* ATCC 25923 was inhibited in the coated surfaces with the funtionalized magnetite nanoparticles over 72 h in comparison with uncoated surfaces. Together, the studies performed by Grumezescu et al. [165] and Iordache et al. [166] demonstrated that the MAPLE method of formulation for nanostructured bioactive surfaces can afford an efficient way to deliver antimicrobial molecules in a controlled mode inhibiting the ability of bacterial pathogens to form biofilm.

**Figure 2:**
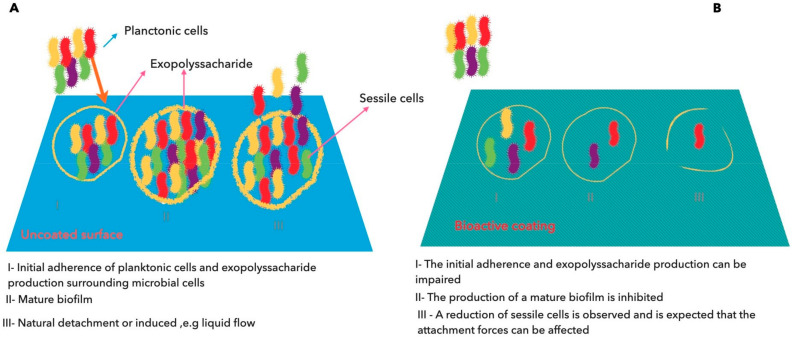
Inhibition of biofilm formation by nanostructured bioactive coating surfaces. (**A**) The uncoated surface allows the adherence of planktonic cells (cells in suspension) with the subsequent production of exopolysaccharides (or glycocalyx) (I) that enclose the sessile cells (adherent cells), forming a mature biofilm (II) and at a later stage can detach from the aggregate and initiate a new cycle of colonization of the same or new surfaces (III). (**B**) A bioactive coating allows the inhibition of the first stage of the biofilm formation: the bacterial adherence and the production of glycocalyx (I). Sessile cells are not able to multiplicate inside the aggregate and the layer of glycocalyx is thinner (II), resulting in a deficient mature biofilm that will collapses (III).

The effect of bioactive surfaces on the inhibition of biofilm formation is illustrated in Figure 2.

## 7. Conclusions

Diverse methods have been developed for producing magnetite nanoparticles with adequate size, shape, size distribution, surface, and magnetic properties. These methodologies almost always integrate functionalization with inorganic or organic molecules for stabilizating the magnetic nanoparticles as a magnetic colloidal suspension, which is particularly important if the medical application is envisaged. Essential oils have been used for the production of magnetite nanoparticles by the sol–gel method combined with autoignition, for modifying the surface magnetite nanoparticles for improving their stabilization and, particularly, to prevent the microbial growth.

The nanobiosystems tested using magnetite nanoparticles with different surfactants and associated with diverse essential oils were able not only to inhibit the adherence process but also the formation of biofilm in different microbial cells. Nevertheless, the utilization of essential oils with complex chemical composition, with dozens of compounds, makes it difficult to attrribute the antimicrobial and/or anti-adherence properties to some of those compounds. Moreover, the chemical composition of some essential oils was not provided. In addition, the diverse techniques used not only for the production of SPIONs, but also for their functionalization with diverse types of polymers without any apparent connection among them make it difficult to compare results and draw conclusions. In addition, there was a limitation of the majority of the studies, which was the use of a sole approach on the evaluation of the formation of biofilm that was mainly comprised by the determination of the microbial cells viability, and other aspects, such as the inhibition of exopolyssacharide production, inhibition of the cell–cell communication, and virulence potential were not covered. It is recognized that all these features can have a tremendous impact on the infection process starting from the release of the surface colonization. Considering this, it is important that future studies include the exploitation of these aspects in order to have a more complete picture about the impact of the nanobiosystems on the associated physiological and virulence potential linked with biofilm.

## Figures and Tables

**Table 1:** Comparison of representative synthesis methods.

Method	Advantages	Disadvantages	Size and Size Distribution	Morphology
Coprecipitation	Large quantities can be synthesized. Simple experimental procedure.	Limited control over the size distribution. Possible oxidation of magnetite to maghemite.	Typically below 50 nm, with broad size distribution.	Spherical with aggregates.
Microemulsion	Good control over the size and shape of the nanoparticles. Low temperature of synthesis.	Limited quantities produced. Use of organic solvents and surfactants that can be difficult to remove.	Usually below 15–20 nm with very narrow size distribution.	Spherical with no aggregates.
High temperature	Very good control over the size, shape, and size distribution of the nanoparticles.	Need high-temperature equipment and, depending on the method, metal organic precursors could be used.	Variable with the method and the precursor. Very small particles can be prepared (ca. 2–3 nm). Very narrow size distribution.	Very different shapes can be prepared, including unusual morphologies as nanopolyhedra, core–shell structures, aggregate nanoflowers, hollow nanoparticles, nanocapsules.
Sol–gel	Particles of desired shape and length can be synthesized. Adequate for the preparation of core–shell nanoparticles and organic–inorganic composites involving magnetic oxides.	The reactions are performed at low temperature, but further treatments at higher temperature are needed to obtain the final structure. Sol–gel matrix residues may remain in the final products.	Nanoparticles smaller than 20 nm are usually prepared, but larger particles (up to 200 nm) have been reported. Usually narrow size distribution.	Usually spherical. High porosity may be introduced.
Spray pyrolysis	High production rate. Cost-effective. High chemical flexibility. Easy control of process parameters.	Large aggregates could be formed.	Particles up to ca. 700 nm depending on the process parameters.	Usually spherical, but aggregates could have different shapes.
